# Correction: Experimental evolution of diverse *Escherichia coli* metabolic mutants identifies genetic loci for convergent adaptation of growth rate

**DOI:** 10.1371/journal.pgen.1007411

**Published:** 2018-05-29

**Authors:** Thomas P. Wytock, Aretha Fiebig, Jonathan W. Willett, Julien Herrou, Aleksandra Fergin, Adilson E. Motter, Sean Crosson

There are citation errors in S3 Table, S4 Table, S5 Table, S6 Table and S7 Table. There are also labelling errors in S2 Fig, S1 Table, S2 Table and S9 Table. In S2 Table, the sentence above the table is incomplete.

Please view the corrected files below.

## Supporting information

S2 Fig Heatmap of RNA-Seq data for genes regulated by H-NS and σ^s^. Rows and columns correspond to genes and samples, respectively. Genes regulated by H-NS and σ^s^ were selected based on [49], and filtered to remove ribosomal RNA genes and genes absent in strain BW25113. In particular, the *hde* and *gad* genes discussed in the text are highlighted in magenta. RNA-sequencing experiments obtained from the NCBI SRA database are listed in S9 Table. Labels detailing strain and growth condition are color-coded for strains with Δ*hns* (red), Δ*rpoS* (gold), unmutated *rpoBC* (green), and mutant *rpoBC* (black). If not indicated otherwise, the strain genetic background is K12 BW25113 and the growth condition is exponential phase in M9. The dendrograms indicate the relatedness of the transcriptional profiles as measured by the Ward metric. Transcriptional fold changes were measured against their corresponding WT sequencing runs, as indicated in S9 Table. Growth phase abbreviations: EE–Early Exponential; ME–Mid-Exponential; TS–Transition to Stationary; S–Stationary; LS–Late Stationary.
(TIF)

S1 Table Specific growth rate and genetic selection information for *E*. *coli* strains grown on M9G. Data are reported for *E*. *coli* BW25113 (WT); five primary mutant strains (Δ*zwf*, Δ*ppk*, Δ*dapF*, Δ*entC*, and Δ*dgk*); 16 independent *sup* strains (which rescue slow growth of metabolic mutants); 16 independent knock-in strains in which the primary mutation was restored to the WT allele (noted in table as ‘restore’); and 12 *fast* strains evolved from WT paired with a repeat of wild-type from paired growth experiments.
(DOCX)

S2 Table LB-cultivated growth rate data. Specific growth rate (in 1/h) is reported for the strains enumerated in S1 Table cultivated in LB.
(DOCX)

S3 Table Analysis of the transcriptomic response to the *dapF* deletion and adaptive evolution. (XLSX)

S4 Table Analysis of the transcriptomic response to the *dgk* deletion and adaptive evolution. (XLSX)

S5 Table Analysis of the transcriptomic response to the *entC* deletion and adaptive evolution. (XLSX)

S6 Table Analysis of the transcriptomic response to the *ppk* deletion and adaptive evolution. (XLSX)

S7 Table Analysis of the transcriptomic response to the *zwf* deletion and adaptive evolution. (XLSX)

S9 Table Description of datasets used in the out-of-sample KNN analysis and prediction performance of our measured growth rates using our RNA-Seq data as input. Tab “Training Data Description” defines the GEO Accession numbers composing each dataset listed in the column titled “Set Name”. Tab “Out of Sample Performance” lists summary predictive performance as measured by the coefficient of determination (R^2^) and the number of experiments predicted within either 25% or 10% accuracy. The size of the dataset used to measure correlations and train the model is detailed in the column “No. Experiments (with Growth)”. The final column lists the number of experiments predicted within the specified accuracy under the leave-one-strain-out model.
(XLSX)
