# Supplementary figures and images for: Experimental evolution of diverse Escherichia coli metabolic mutants identifies genetic loci for convergent adaptation of growth rate
Source: PLoS Genet. 2018 Mar 27;14(3):e1007284. doi: 10.1371/journal.pgen.1007284 (PMC5892946; doi:10.1371/journal.pgen.1007284)

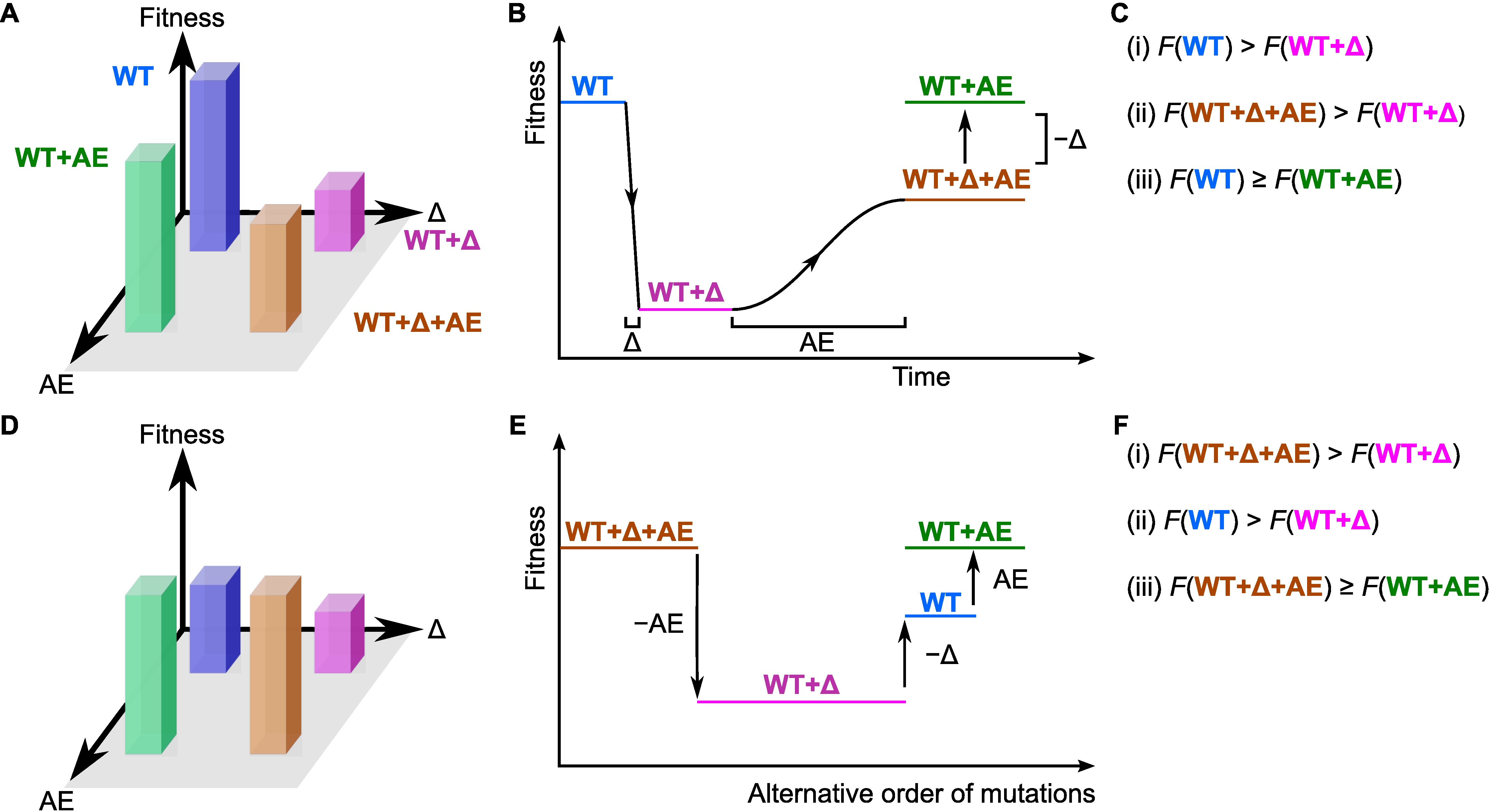

Supplement: S1 Fig — (A) Bar graph of fitness (i.e., growth rate) illustrating synthetic rescue epistasis in which a gene deletion (Δ) is rescued by adaptively evolved sup mutations. The fitness of the WT strain (blue) decreases upon gene deletion (WT + Δ, pink) and increases upon acquisition of sup mutations during adaptive evolution (AE) (WT + Δ + AE, orange). The impact of sup mutations acquired during AE on the WT strain (WT + AE, green) is neutral at best. (B) Order of the mutations in (A), where the WT + AE strain is constructed by restoring the primary gene deletion in the sup strain. (C) Mathematical conditions defining synthetic rescues, where F is the fitness of each genetic background. (D, E) A posteriori approach to identify synthetic rescue interactions, considering an alternative order of mutations. The initial strain is WT + Δ + AE; genetic perturbations are the restoration of the sup mutations acquired through AE (–AE) and the restoration of the primary gene deletion mutation (–Δ). (F) Counterpart of (C) for the alternative order in (D, E). Specific examples of synthetic rescues from our experiments are presented in Table 3 for both mutational orders. (TIF) [file pgen.1007284.s001.tif]

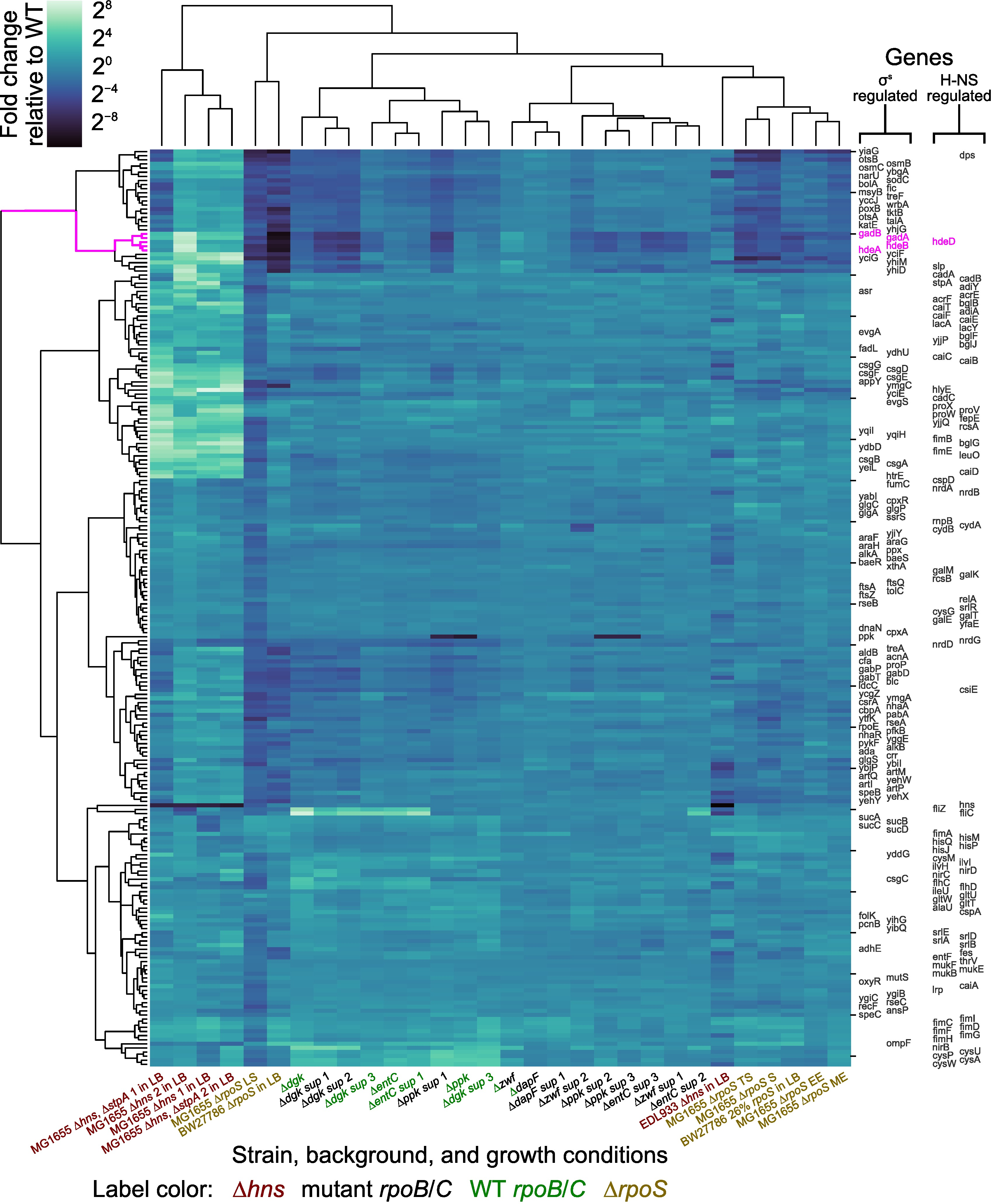

Supplement: S2 Fig — Rows and columns correspond to genes and samples, respectively. Genes regulated by H-NS and σs were selected based on [49], and filtered to remove ribosomal RNA genes and genes absent in strain BW25113. In particular, the hde and gad genes discussed in the text are highlighted in magenta. RNA-sequencing experiments obtained from the NCBI SRA database are listed in S9 Table. Labels detailing strain and growth condition are color-coded for strains with Δhns (red), ΔrpoS (gold), unmutated rpoBC (green), and mutant rpoBC (black). If not indicated otherwise, the strain genetic background is K12 BW25113 and the growth condition is exponential phase in M9. The dendrograms indicate the relatedness of the transcriptional profiles as measured by the Ward metric. Transcriptional fold changes were measured against their corresponding WT sequencing runs, as indicated in S9 Table. Growth phase abbreviations: EE–Early Exponential; ME–Mid-Exponential; TS–Transition to Stationary; S–Stationary; LS–Late Stationary. (TIF) [file pgen.1007284.s002.tif]
