# Supplementary material for: Experimental evolution of diverse Escherichia coli metabolic mutants identifies genetic loci for convergent adaptation of growth rate
Source: PLoS Genet. 2018 Mar 27;14(3):e1007284. doi: 10.1371/journal.pgen.1007284 (PMC5892946; doi:10.1371/journal.pgen.1007284)
Supplement: S2 Table — Specific growth rate (in 1/h) is reported for the strains enumerated in S1 Table cultivated in LB. (DOCX) [file pgen.1007284.s004.docx]

**S2 Table.** Growth rate data for LB-cultivated wild-type *E. coli* and *Fast* strains, the five primary mutant strains (Δ*zwf*, Δ*ppk*, Δ*dapF*, Δ*entC*, Δ*dgk*), 16 independent suppressor (*sup*) strains, and 16 “knock-in” strains with the restored primary mutant.

| **Strain** | **Mean population growth rate**  **(1/h ± SD)** | **Final fitness**  **(wild type = 1.00)** |
| --- | --- | --- |
| E. coli K-12 BW25113 | 1.64 ± 0.08 | 1.00 |
| E. coli Δzwf | 1.12 ± 0.02 | 0.68 |
| E. coli Δzwf sup1 | 1.21 ± 0.04 | 0.74 |
| E. coli Δzwf sup2 | 0.92 ± 0.03 | 0.56 |
| E. coli Δzwf sup3 | 1.18 ± 0.05 | 0.72 |
| E. coli Δzwf sup4 | 1.15 ± 0.07 | 0.70 |
| zwf restore-sup1 | 1.52 ± 0.10 | 0.93 |
| zwf restore-sup2 | 0.90 ± 0.05 | 0.55 |
| zwf restore-sup3 | 1.62 ± 0.03 | 0.99 |
| zwf restore-sup4 | 1.60 ± 0.08 | 0.98 |
| E. coli Δppk | 1.64 ± 0.06 | 1.00 |
| E. coli Δppk sup1 | 1.49 ± 0.05 | 0.91 |
| E. coli Δppk sup2 | 1.55 ± 0.03 | 0.95 |
| E. coli Δppk sup3 | 1.53 ± 0.08 | 0.93 |
| ppk restore-sup1 | 1.55 ± 0.06 | 0.95 |
| ppk restore-sup2 | 1.54 ± 0.04 | 0.94 |
| ppk restore-sup3 | 1.51 ± 0.10 | 0.92 |
| E. coli ΔdapF | 0.95 ± 0.07 | 0.58 |
| E. coli ΔdapF sup1 | 1.17 ± 0.06 | 0.71 |
| E. coli ΔdapF sup2 | 1.19 ± 0.06 | 0.73 |
| E. coli ΔdapF sup3 | 1.04 ± 0.05 | 0.63 |
| dapF restore-sup1 | 1.13 ± 0.06 | 0.69 |
| dapF restore-sup2 | 1.13 ± 0.03 | 0.69 |
| dapF restore-sup3 | 1.04 ± 0.07 | 0.63 |
| E. coli ΔentC | 1.67 ± 0.07 | 1.02 |
| E. coli ΔentC sup1 | 1.56 ± 0.10 | 0.95 |
| E. coli ΔentC sup2 | 1.74 ± 0.08 | 1.06 |
| E. coli ΔentC sup3 | 1.63 ± 0.08 | 0.99 |
| entC restore-sup1 | 1.58 ± 0.03 | 0.96 |
| entC restore-sup2 | 1.79 ± 0.05 | 1.09 |
| entC restore-sup3 | 1.70 ± 0.08 | 1.04 |
| E. coli Δdgk | 1.61 ± 0.05 | 0.98 |
| E. coli Δdgk sup1 | 1.50 ± 0.06 | 0.91 |
| E. coli Δdgk sup2 | 1.43 ± 0.07 | 0.87 |
| E. coli Δdgk sup3 | 1.66 ± 0.05 | 1.01 |
| dgk restore-sup1 | 1.56 ± 0.07 | 0.95 |
| dgk restore-sup2 | 1.64 ± 0.10 | 1.00 |
| dgk restore-sup3 | 1.77 ± 0.02 | 1.08 |
|  |  |  |
| E. coli K-12 BW25113 | 1.85 ± 0.05 | 1.00 |
| Fast_1a | 1.70 ± 0.03 | 0.92 |
| Fast_1b | 1.72 ± 0.07 | 0.93 |
| Fast_2a | 1.68 ± 0.10 | 0.91 |
| Fast_2b | 1.71 ± 0.08 | 0.93 |
| Fast_3a | 1.73 ± 0.04 | 0.94 |
| Fast_3a | 1.71 ± 0.10 | 0.93 |
| Fast_4a | 1.89 ± 0.08 | 1.02 |
| Fast_4b | 1.85 ± 0.12 | 1.00 |
| Fast_5a | 1.76 ± 0.09 | 0.95 |
| Fast_5b | 1.78 ± 0.08 | 0.96 |
| Fast_6a | 1.37 ± 0.04 | 0.74 |
| Fast_6b | 1.35 ± 0.03 | 0.73 |
