# Supplementary figures and images for: Correction: Experimental evolution of diverse Escherichia coli metabolic mutants identifies genetic loci for convergent adaptation of growth rate
Source: PLoS Genet. 2018 May 29;14(5):e1007411. doi: 10.1371/journal.pgen.1007411 (PMC5973558; doi:10.1371/journal.pgen.1007411)

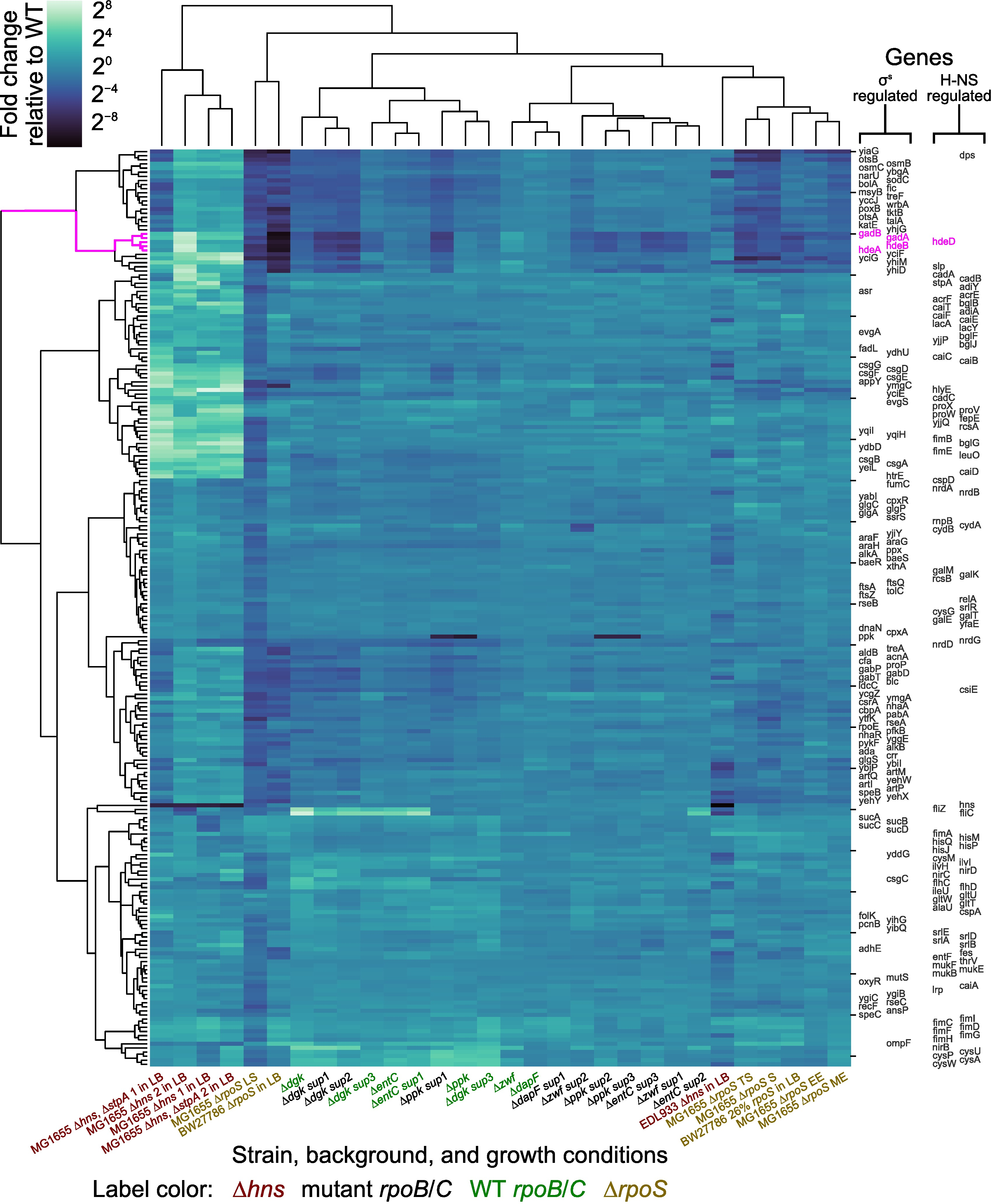

Supplement: S2 Fig — Rows and columns correspond to genes and samples, respectively. Genes regulated by H-NS and σs were selected based on [49], and filtered to remove ribosomal RNA genes and genes absent in strain BW25113. In particular, the hde and gad genes discussed in the text are highlighted in magenta. RNA-sequencing experiments obtained from the NCBI SRA database are listed in S9 Table. Labels detailing strain and growth condition are color-coded for strains with Δhns (red), ΔrpoS (gold), unmutated rpoBC (green), and mutant rpoBC (black). If not indicated otherwise, the strain genetic background is K12 BW25113 and the growth condition is exponential phase in M9. The dendrograms indicate the relatedness of the transcriptional profiles as measured by the Ward metric. Transcriptional fold changes were measured against their corresponding WT sequencing runs, as indicated in S9 Table. Growth phase abbreviations: EE–Early Exponential; ME–Mid-Exponential; TS–Transition to Stationary; S–Stationary; LS–Late Stationary. (TIF) [file pgen.1007411.s001.tif]
