# Supplementary material for: Correction: Experimental evolution of diverse Escherichia coli metabolic mutants identifies genetic loci for convergent adaptation of growth rate
Source: PLoS Genet. 2018 May 29;14(5):e1007411. doi: 10.1371/journal.pgen.1007411 (PMC5973558; doi:10.1371/journal.pgen.1007411)
Supplement: S1 Table — Data are reported for E. coli BW25113 (WT); five primary mutant strains (Δzwf, Δppk, ΔdapF, ΔentC, and Δdgk); 16 independent sup strains (which rescue slow growth of metabolic mutants); 16 independent knock-in strains in which the primary mutation was restored to the WT allele (noted in table as ‘restore’); and 12 fast strains evolved from WT paired with a repeat of wild-type from paired growth experiments. (DOCX) [file pgen.1007411.s002.docx]

**S1 Table.** Growth rate data and genetic selection information for *Escherichia coli* strains, grown on M9 medium supplemented with glucose.

| **Strain** | **Mean population growth rate**  **(1/h ± SD)** | **Estimated total doublings (range)** | **Final fitness**  **(WT = 1.00)** | **Number of serial transfers** |
| --- | --- | --- | --- | --- |
| E. coli K-12 BW25113 | 0.62 ± 0.03 | NA | 1.00 | NA |
| E. coli Δzwf | 0.57 ± 0.03 | NA | 0.91 | NA |
| E. coli Δzwf sup1 | 0.73 ± 0.01 | 265–510 | 1.18 | 56 |
| E. coli Δzwf sup2 | 0.72 ± 0.02 | 265–341 | 1.16 | 56 |
| E. coli Δzwf sup3 | 0.70 ± 0.02 | 265–498 | 1.13 | 56 |
| E. coli Δzwf sup4 | 0.74 ± 0.02 | 265–497 | 1.20 | 56 |
| zwf restore-sup1 | 0.71 ± 0.01 | NA | 1.15 | NA |
| zwf restore-sup2 | 0.70 ± 0.02 | NA | 1.12 | NA |
| zwf restore-sup3 | 0.69 ± 0.03 | NA | 1.11 | NA |
| zwf restore-sup4 | 0.75 ± 0.01 | NA | 1.21 | NA |
| E. coli Δppk | 0.61 ± 0.02 | NA | 0.99 | NA |
| E. coli Δppk sup1 | 0.76 ± 0.01 | 334–590 | 1.23 | 56 |
| E. coli Δppk sup2 | 0.77 ± 0.01 | 334–586 | 1.24 | 56 |
| E. coli Δppk sup3 | 0.75 ± 0.01 | 334–562 | 1.21 | 56 |
| ppk restore-sup1 | 0.76 ± 0.01 | NA | 1.23 | NA |
| ppk restore-sup2 | 0.76 ± 0.01 | NA | 1.23 | NA |
| ppk restore-sup3 | 0.74 ± 0.02 | NA | 1.20 | NA |
| E. coli ΔdapF | 0.19 ± 0.03 | NA | 0.31 | NA |
| E. coli ΔdapF sup1 | 0.66 ± 0.02 | 216–482 | 1.06 | 56 |
| E. coli ΔdapF sup2 | 0.64 ± 0.03 | 216–494 | 1.04 | 56 |
| E. coli ΔdapF sup3 | 0.66 ± 0.02 | 216–497 | 1.06 | 56 |
| dapF restore-sup1 | 0.74 ± 0.03 | NA | 1.19 | NA |
| dapF restore-sup2 | 0.68 ± 0.02 | NA | 1.10 | NA |
| dapF restore-sup3 | 0.76 ± 0.02 | NA | 1.22 | NA |
| E. coli ΔentC | 0.29 ± 0.04 | NA | 0.47 | NA |
| E. coli ΔentC sup1 | 0.70 ± 0.02 | 232–478 | 1.13 | 42 |
| E. coli ΔentC sup2 | 0.78 ± 0.01 | 232–534 | 1.26 | 42 |
| E. coli ΔentC sup3 | 0.83 ± 0.02 | 232–468 | 1.33 | 42 |
| entC restore-sup1 | 0.76 ± 0.02 | NA | 1.23 | NA |
| entC restore-sup2 | 0.85 ± 0.02 | NA | 1.37 | NA |
| entC restore-sup3 | 0.86 ± 0.02 | NA | 1.39 | NA |
| E. coli Δdgk | 0.60 ± 0.01 | NA | 0.96 | NA |
| E. coli Δdgk sup1 | 0.83 ± 0.03 | 452–594 | 1.34 | 42 |
| E. coli Δdgk sup2 | 0.82 ± 0.02 | 452–611 | 1.32 | 42 |
| E. coli Δdgk sup3 | 0.77 ± 0.02 | 452–587 | 1.24 | 42 |
| dgk restore-sup1 | 0.86 ± 0.01 | NA | 1.38 | NA |
| dgk restore-sup2 | 0.86 ± 0.01 | NA | 1.38 | NA |
| dgk restore-sup3 | 0.82 ± 0.01 | NA | 1.32 | NA |
|  |  |  |  |  |
| E. coli K-12 BW25113 | 0.60 ± 0.01 | NA | 1.00 | NA |
| Fast_1a | 0.78 ± 0.02 | 160-240 | 1.30 | 24 |
| Fast_1b | 0.78 ± 0.02 | 160-240 | 1.29 | 24 |
| Fast_2a | 0.76 ± 0.003 | 160-240 | 1.27 | 24 |
| Fast_2b | 0.75 ± 0.02 | 160-240 | 1.25 | 24 |
| Fast_3a | 0.77 ± 0.03 | 160-240 | 1.29 | 24 |
| Fast_3b | 0.78 ± 0.02 | 160-240 | 1.30 | 24 |
| Fast_4a | 0.83 ± 0.04 | 160-240 | 1.38 | 24 |
| Fast_4b | 0.86 ± 0.02 | 160-240 | 1.43 | 24 |
| Fast_5a | 0.77 ± 0.03 | 160-240 | 1.28 | 24 |
| Fast_5b | 0.76 ± 0.03 | 160-240 | 1.27 | 24 |
| Fast_6a | 0.90 ± 0.02 | 160-240 | 1.49 | 24 |
| Fast_6b | 0.87 ± 0.03 | 160-240 | 1.45 | 24 |

Strains used were: wild-type (WT) *Escherichia coli*; five primary mutant strains (Δ*zwf*, Δ*ppk*, Δ*dapF*, Δ*entC*, and Δ*dgk*); 16 independent suppressor (*sup*) strains (which rescue slow growth of metabolic mutants); 16 independent knock-in strains in which the primary mutation was restored to WT (noted in table as ‘restore’); and 12 *Fast* strains evolved from WT paired with a repeat of wild-type from paired growth experiments.
